# Sero-prevalence of arthropod-borne viral infections among Lukanga swamp residents in Zambia

**DOI:** 10.1371/journal.pone.0235322

**Published:** 2020-07-01

**Authors:** Caroline C. Chisenga, Samuel Bosomprah, Kalo Musukuma, Cynthia Mubanga, Obvious N. Chilyabanyama, Rachel M. Velu, Young Chan Kim, Arturo Reyes-Sandoval, Roma Chilengi

**Affiliations:** 1 Centre for Infectious Diseases Research in Zambia, Lusaka, Zambia; 2 Department of Biostatistics, School of Public Health, University of Ghana, Accra; 3 The Jenner Institute, University of Oxford, The Henry Wellcome Building for Molecular Physiology, Oxford, England, United Kingdom; Faculty of Science, Ain Shams University (ASU), EGYPT

## Abstract

**Introduction:**

The re-emergence of vector borne diseases affecting millions of people in recent years has drawn attention to arboviruses globally. Here, we report on the sero-prevalence of chikungunya virus (CHIKV), dengue virus (DENV), mayaro virus (MAYV) and zika virus (ZIKV) in a swamp community in Zambia.

**Methods:**

We collected blood and saliva samples from residents of Lukanga swamps in 2016 during a mass-cholera vaccination campaign. Over 10,000 residents were vaccinated with two doses of Shanchol^™^ during this period. The biological samples were collected prior to vaccination (baseline) and at specified time points after vaccination. We tested a total of 214 baseline stored serum samples for IgG antibodies against NS1 of DENV and ZIKV and E2 of CHIKV and MAYV on ELISA. We defined sero-prevalence as the proportion of participants with optical density (OD) values above a defined cut-off value, determined using a finite mixture model.

**Results:**

Of the 214 participants, 79 (36.9%; 95% CI 30.5–43.8) were sero-positive for Chikungunya; 23 (10.8%; 95% CI 6.9–15.7) for Zika, 36 (16.8%; 95% CI 12.1–22.5) for Dengue and 42 (19.6%; 95% CI 14.5–25.6) for Mayaro. Older participants were more likely to have Zika virus whilst those involved with fishing activities were at greater risk of contracting Chikungunya virus. Among all the antigens tested, we also found that Chikungunya saliva antibody titres correlated with baseline serum titres (Spearman’s correlation coefficient = 0.222; p = 0.03).

**Conclusion:**

Arbovirus transmission is occurring in Zambia. This requires proper screening tools as well as surveillance data to accurately report on disease burden in Zambia.

## Introduction

Chikungunya (CHIKV), dengue (DENV), mayaro (MAYV) and zika (ZIKV) are arthropod-borne viruses [1–3], informally referred to as arboviruses. CHIKV, DENV, and ZIKV are mainly transmitted by the *Aedes aegypti and Aedes albopictus* mosquitoes [4] while MAYV is transmitted via the *Haemogogus* mosquito [1,5–8] although it has been isolated in other mosquito genera such as *Culex*, *Aedes* and *Psorophora* [9]. These viruses have been in circulation for over 65 years and are responsible for many outbreaks and sporadic cases in various countries [10–12]. Infection with any of these viruses may result in febrile illness, arthralgia, fever, headache, joint pain, tiredness, and rash [11].

The re-emergence of vector borne diseases affecting millions of people in recent years has drawn attention to arboviruses globally. The World Health Organisation (WHO), in February 2016, announced a Public Health Emergency of International Concern [12] following ZIKV transmission and a possible link to congenital disorders in the Americas and the Pacific Islands [13–18] which was later suggested as a cause to microcephaly and other congenital disorders [19–21]. Tanzania and Congo DR have reported high occurrence of *Aedes aegypti* and arboviral diseases [22,23]. Yellow fever and dengue have been investigated [24,25] in Zambia.

It is challenging to predict where and when arbovirus outbreaks will occur. Zambia is a malaria-endemic country [26] and since there exists a similarly in the clinical presentation of arbovirus related illnesses to that of malaria, there is the possibility of underdiagnosis of arboviruses [27]. Similar to malaria, CHIKV, MAYV (E2), DENV and ZIKV can also present with joint pain, high fever and headache among others [28], therefore, it remains unclear to what extent people are generally exposed [29–31]. Data on the fraction of the population susceptible to infection could provide some clue to potential epidemic. Serological surveys in which antibody responses to a pathogen are measured, allow direct measurement of the population that remain susceptible for future infection. In this study, we sought to estimate sero-prevalence of arboviruses among Lukanga swamps residents in Zambia. In an exploratory scenario, we postulated that saliva can be an alternative, less invasive and cheaper option for future surveillance studies to support the assessment of disease burden. Therefore, this study also aimed to establish whether there exists a correlation of antibodies against arboviruses in both saliva and serum.

## Methods

### Study area

The study was conducted in Lukanga swamps, which is located 70 km northwest of the capital city of Central Province, Kabwe and 130 km from Kapiri Mposhi District. The swamps are shared among the six districts of Central Province namely Kabwe, Mumbwa, Ngabwe, Chisamba, Chibombo and Kapiri Mposhi. The swamps are easily accessible through Kabwe and fall within the Waya Health Centre (WHC) catchment area of Kapiri Mposhi district. Waya Health Centre has a catchment population of 21,000, with 16,000 living on the upper land, and 5000 living within the swamps [32]. The majority of the population depends on fishing activities for their livelihood [33].

### Study design and participants

We screened/reviewed blood and saliva samples collected among fishmongers in Lukanga swamps, Central Province of Zambia at WHC from October to November 2016. Fishmongers are male and females who fish and trade in fishing, live within Lukanga swamps. They spend long periods of time (months) in the swamps, an environment which is very favourable for sustaining mosquitoes and this pre-disposes them to mosquito bites. We tested all baseline left over serum samples (~500μl) collected during cholera vaccine trial. In the trial, participants were considered eligible if they were aged 18 years and above, resided in Lukanga swamps for over a year, involved with fish mongering, willing to be followed up for 2 years, willing to be vaccinated, healthy by medical history and clinical examination, and willing and able to provide written informed consent. Pregnant women were excluded as well as those with fever, any acute disease, any systemic disorder as determined by medical history or physical examination that would compromise the participant’s health.

### Procedures

Approximately 10 ml of venepunture bloods in EDTA tubes and 1 ml saliva samples were collected at baseline during the mass-cholera vaccination campaign in Lukanga swamps in 2016 in which over 10,000 residents were vaccinated with two doses of Shanchol^™^ (Shantha Biotechnics, Ranga Reddy District, Telangana, India, a subsidiary of the French pharmaceutical company Sanofi-Aventis). Biological samples were collected prior to vaccination and at specified time points, that is baseline, day 28, 6, 12, 18, 24, 30, 36, 42, and 48 months. In order to test for antibodies against circulating arboviruses, we developed an enzyme-linked immunosorbent assay (ELISA) based on antigens specific to CHIKV and MAYV (E2), and DENV and ZIKV (NS1) arboviruses, which have been used to study patients from endemic regions of Mexico previously [34]. All serum samples collected at baseline and saliva samples from the same individuals collected a year later during follow up were subjected to the ELISA assay. All participants enrolled in the parent study gave consent by signing the informed consent form and copy was given to them for further reference. However, for this study, since we could not go back and re-consent the participants, a waver on re-consenting was granted by the University of Zambia Biomedical Research Authority, Lusaka, Zambia.

After 10 mls of the blood draw in EDTA tubes, samples were immediately centrifuged at 3000 rpm for 20 minutes to separate ~4 mls of serum for storage at -80°C in two separate aliquotes. Saliva was collected using Salimetric swabs into SalivaBio Swab tubes (Salimetrics, USA) and was transported to the laboratory in cool box with ice packs. To SalivaBio Swab tubes containing a Salimetrics swab, 1 mL of antibody transport medium (containing 0.2% Tween 20, 10% foetal bovine serum (FBS) and 0.7% antibiotic/anti-mycotic, in phosphate-buffered saline, pH 7.2) was added, vortexed for 20 seconds and centrifuged at 3000 rpm for about 10 minutes. After centrifugation, the processed saliva was recovered and stored at -80°C until testing for salivary arboviral antibody, IgG.

The ELISA assay on both baseline serum samples and saliva colleted at 1 year was conducted as described previously [34,35]. Briefly, plates were coated with 2 μg/mL of antigen diluted in PBS (coating buffer). 50 μL of the coating buffer was added to each well. All coated plates remained at room temperature (RT) overnight and the next day, the plates were washed 6 times with PBS/0.05% Tween (PBS/Tween). The plates were then blocked by adding 300 μL of Pierce Blocking Buffer to each well and allowing the plates to sit for 2h at RT. Sera were diluted 1:200 in PBS/Tween by adding 5 μl sera to 995 μl PBS/T. Previously tested positive sera was used as positive control and PBS/Tween as a blank. After a 2 hour incubation, blocking buffer was discarded and the plates were washed 6 times with PBS/0.05% Tween (PBS/Tween). 75 μL of the diluted sera were added to the wells in duplicate after which the plates were further incubated at RT for 1 hour and later washed 6 times with PBS/Tween.

50 μL of Detecting Ab–Anti-Human IgG-alkaline phosphatase-conjugated antibody (A3187-5ML) diluted 1/5000 in PBS/T (5 mL per plate + 1 μL Ab) was added to each well and the plates incubated at RT for 1 hour. During this time, the substrate, para-Nitrophenylphosphate (pNPP) was prepared by dissolving a 20 mg pNPP tablet in 20 mL of 1X diethanolamine buffer in the dark at RT. 10 mL was required per plate. After the 1 hour incubation, plates were washed 6 times with PBS/Tween and then 100 μL of the previously prepared pNPP substrate was added to each well. The plates were incubated in the dark for 40 minutes at RT and antibody detection read at 405nm on a BioTeck Microplate Reader using Gen5 software. For the exploratory assay, although serum gives a definitive titres reading, saliva and serum samples were paired and diluted 1:5 by adding 20 μl saliva to 80μl PBS/Tween. The rest of the assay steps were similar to that of serum.

Since our laboratory was setting up this type of an ELISA for the first time, we run external quality assurance testing at the National Malaria Control Centre (NMCC) in Lusaka Zambia. The NMCC has set up the qualitative, multiplex, polymerase chain reaction (PCR) based Luminex x-TAG^®^ (Luminex Corporation, Austin TX, USA) for arboviruses. We selected two positive and two negative samples for testing on this platform. Using the arbovirus based PCR on Luminex, three of our samples (one high postive sample for Chikungunya, Zika and Mayaro arboviruses and two negatives for all three) were correctly identified as reported on ELISA. One sample that was moderately positive on ELISA was categorised as negative.

### Outcomes

The primary outcome was sero-prevalence of antibodies against arboviruses. The secondary outcome was correlation of specific IgG antibodies in saliva with serum.

### Statistical analysis

We calculated sample size of 173 using cochrane’s formular based on reported prevalence of Chingunya virus of 12.9% [36], 95% confidence interval and precision of ± 5%. We inflated our sample size by a factor of 24% to account for contamination of blood samples, thus our sample size was 214.

Social demographic characteristics of the participants were summarised using frequencies. Violin plots were used to visualise the distribution of raw O.D measurements of antibodies for each virus. We defined sero-prevalence as the proportion of participants with OD values above a certain cut-off value, determined using finite mixture model. We assumed that the OD values were a mixture of two lognormal distributions. One of the two distributions represents sero-negative population and the other sero-positive population. We defined the cut off as the mean log_10_OD of the log-normal distribution of the sero-negative population plus three standard deviation. We used Pearson’s chi-square or Fisher’s exact tests as appropriate to assess association of sero-prevalence with social demographic characteristics. We used Peasrson’s correlation to test for the strength of association between; serum OD values and saliva OD values and OD values of each virus against other viruses. A p-value of 0.05 or less was considered to be statistically significant. All statistical analyses were performed in Stata 16 MP2 (StataCorp, College Station, TX, USA).

### Ethical approval

Ethical approval for this study was approved through the University of Zambia Biomedical Research Ethics Committee (Ref: 003-04-19), while the National Health Research Authority provided the authorisation to conduct the study. The Zambia Medicines Regulatory Authority were equally notified about the study. Written informed consent prior to initiation of study procedures was sought from all participants in the parent study.

## Results

### Characteristics of participants

A total of 225 participants were recruited into the main study from October through November 2016. All gave consent for participating in the study during the massive vaccination against cholera in the Lukanga swamps. Of these, 214 serum samples were available for testing IgG antibodies against CHIKV, DENV, MAYV, and ZIKV arboviruses. Out of 214, 75% were male, 25% were aged between 36–45 years (Table 1).

**Table 1:** Sero-prevalence of antibody responses to aboviruses by baseline characteristics of participants.

	n(%) of total	Chikungunya		Zika		Dengue		Mayaro	
		n(%) positive	95% CI	n(%) positive	95% CI	n(%) positive	95% CI	n(%) positive	95% CI
**Age (n = 213)**									
< 24 years	38(17.8)	11(29.0)	15.4–45.9	0(0.0)	0–9.3	6(15.8)	6–31.3	11(29.0)	15.4–45.9
24–35 years	58(27.2)	20(34.5)	22.5–48.1	8(13.8)	9.6–37.3	11(19.0)	9.9–31.4	10(17.2)	8.6–29.4
36–45 years	66(31.0)	25(37.9)	26.2–50.7	7(10.6)	7.7–34.3	10(15.2)	7.5–26.1	11(16.7)	8.6–27.9
45+ years	51(23.9)	22(43.1)	29.3–57.8	8(15.7)	9.6–37.3	9(17.7)	8.4–30.9	9(17.7)	8.4–30.9
**Gender**									
Female	18(8.41)	4(22.2)	6.4–47.6	0(0.0)	0–18.5	2(11.1)	1.4–34.7	2(11.1)	1.4–34.7
Male	196(91.59)	75(38.3)	31.4–45.5	23(11.7)	7.6–17.1	34(17.4)	12.3–23.4	40(20.4)	15–26.7
**Ocuppation**									
Farmer	8(3.7)	3(37.5)	17.12–75.5	1(12.5)	0.3–52.7	2(25.0)	3.2–65.1	0(0.0)	0–36.9
Fisher man/woman	148(69.2)	63(42.6)	34.49–51	20(13.5)	8.5–20.1	26(17.6)	11.8–24.7	33(22.3)	15.9–29.9
Trader	38(17.8)	7(18.4)	7.74–34.3	1(2.6)	0.1–13.8	7(18.4)	7.7–34.3	7(18.4)	7.7–34.3
Other	20(9.4)	6(30.0)	11.89–54.3	1(5.0)	0.1–24.9	1(5.0)	0.1–24.9	2(10.0)	1.2–31.7
**Blood group**									
A+	56(26.2)	21(37.5)	24.92–51.5	7(12.5)	5.2–24.1	10(17.9)	8.9–30.4	12(21.4)	11.6–34.4
AB+	7(3.3)	1(14.3)	0.36–57.9	1(14.3)	0.4–57.9	0(0)	0–41	1(14.3)	0.4–57.9
B+	43(20.1)	15(34.9)	21.01–50.9	2(4.7)	0.6–15.8	12(27.9)	15.3–43.7	9(20.9)	10.0–36.0
B-	5(2.3)	0(0.0)	0–52.2^1^	0(0.0)	0–52.2	0(0.0)	0–52.2	0(0.0)	0–52.2
O+	99(46.3)	39(39.4)	29.72–49.7	13(13.1)	7.2–21.4	14(14.1)	8–22.6	19(19.2)	12–28.3
O-	4(1.9)	3(75.0)	19.41–99.4	0(0)	0–60.2	0(0.0)	0–60.2	1(25.0)	0.6–80.6
**Total**	**214**	**79(36.9)**	**30.5–43.8**	**23(10.8)**	**6.9–15.7**	**36(16.8)**	**12.1–22.5**	**42(19.6)**	**14.5–25.6**

### Sero-prevalence among participants

**Fig 1:**
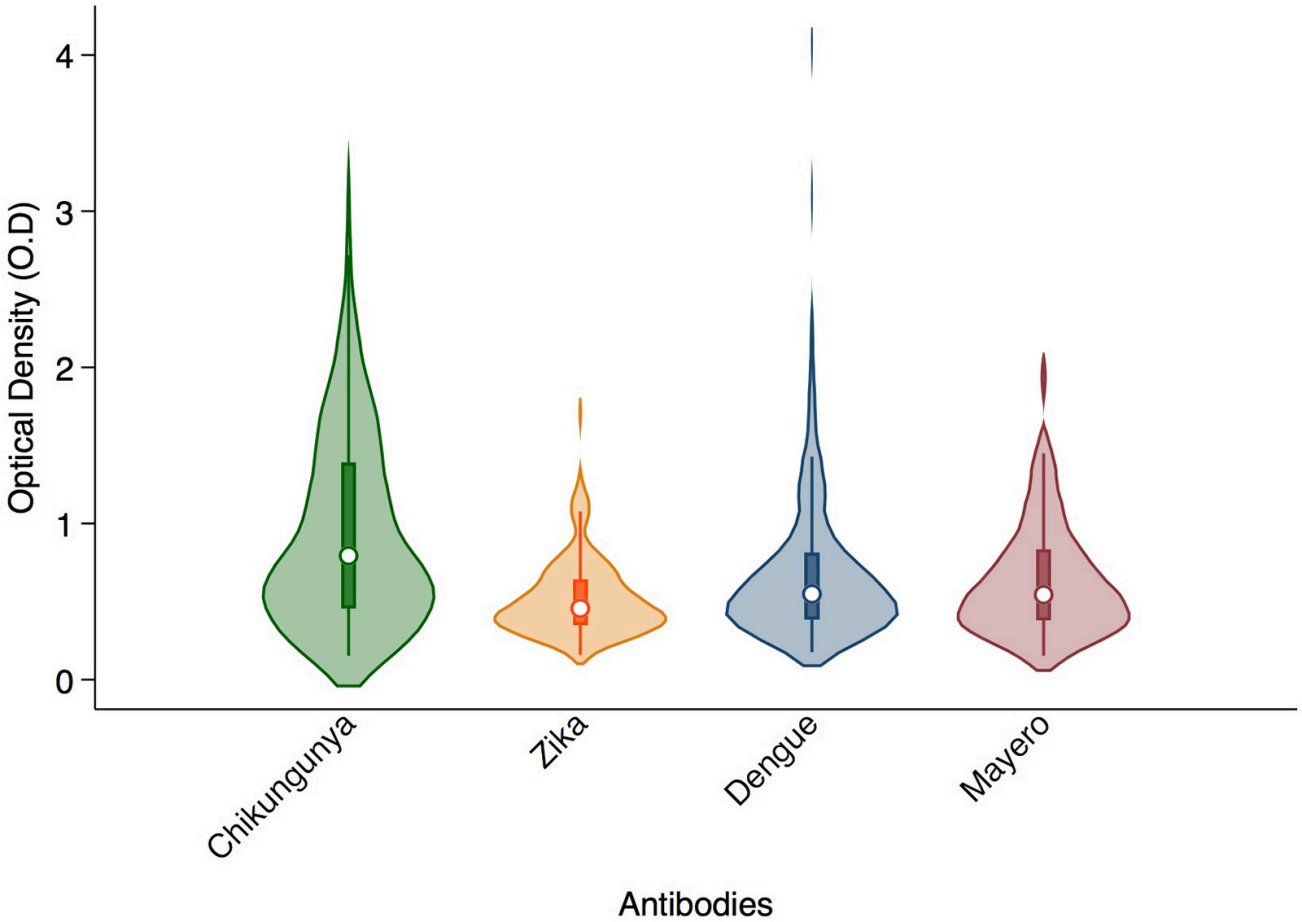
Violin Plot showing the median and distribution for Chikungunya, Zika, Dengue and Mayaro.

**Fig 2:**
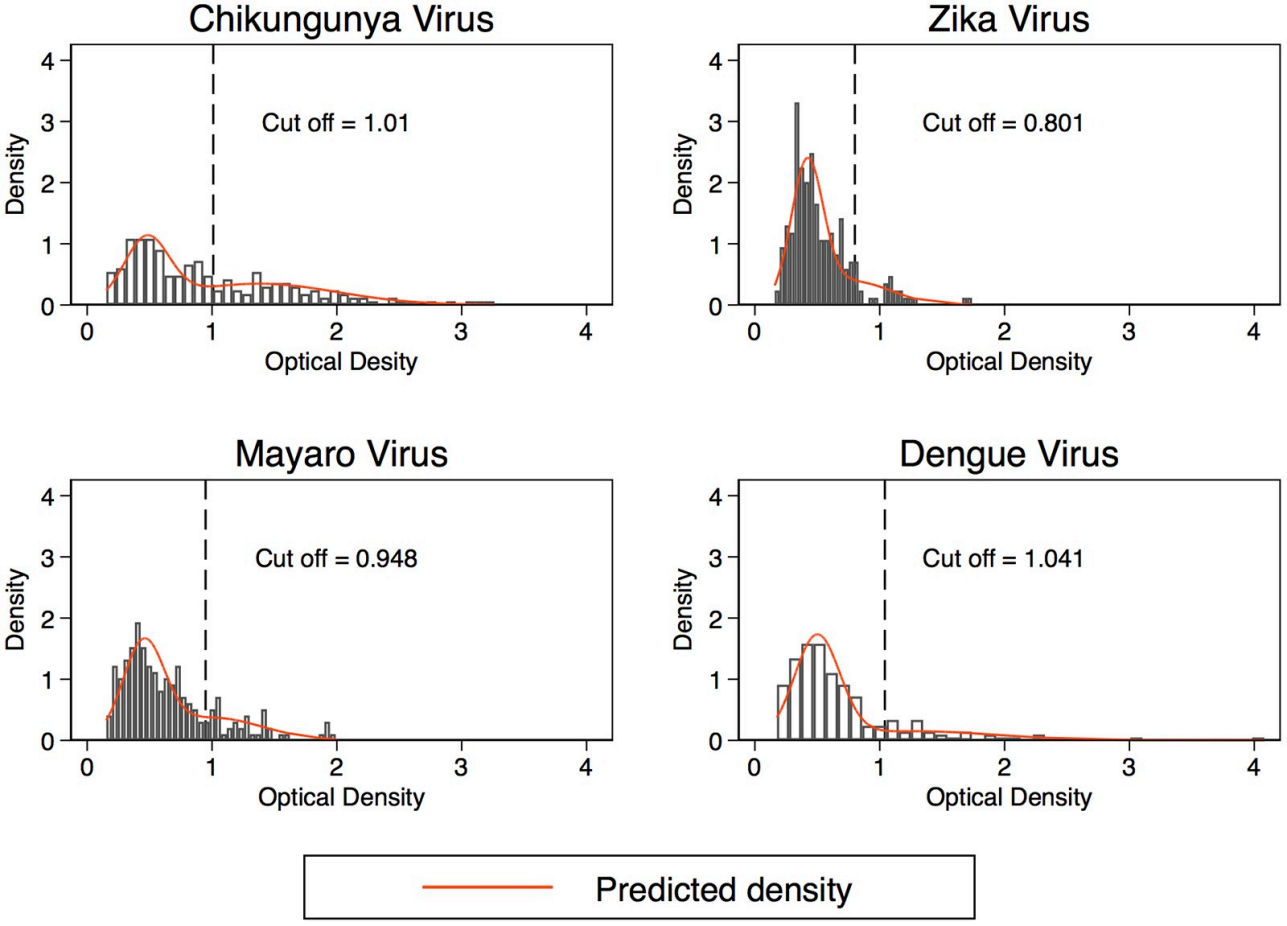
Distribution of ODs for each arboviruses. Vertical lines indicate the cut-off value using finite mixture models at mean OD+3SD of lower OD distribution.

The median (IQR) optical density was 0.79(0.45–1.39), 0.46(0.35–0.64), 0.55(0.38–0.811), and 0.54(0.38–0.83) for Chikungunya, Zika, Dengue, and Mayaro viruses respectively (Fig 1). Of the 214 participants included in the analysis, 79 (36.9%; 95% CI 30.5–43.8) were sero-positive for Chikungunya; 23 (10.8%; 95% CI 6.9–15.7) for Zika, 36 (16.8%; 95% CI 12.1–22.5) for Dengue and 42 (19.6%; 95% CI 14.5–25.6) for Mayaro, (Table 1). The cut-off for sero-prevalence were 1.01, 0.80, 0.94 and 1.0 for Chikungunya, Zika, Mayaro and Dengue viruses, respectively (Fig 2).

### Correlation of specific IgG antibodies in saliva with serum

**Fig 3:**
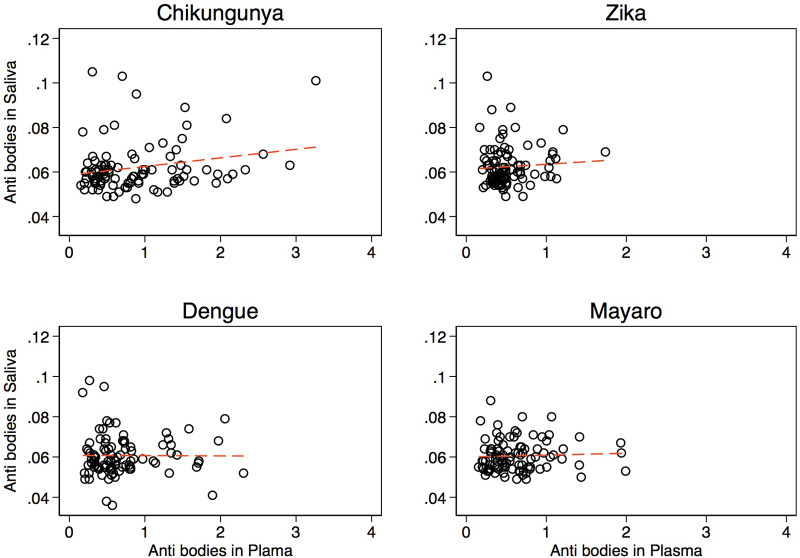
Correlation of anti-arbovirus antibodies between serum and saliva.

For our saliva exploratory assay, we found that individuals with very high antibody titres in serum were likely to have detectable amounts of antibodies in saliva (Fig 3). However, when analysed statistically, very weak correlations were found between anti-arbovirus antibodies in serum and saliva (Fig 3).

**Fig 4:**
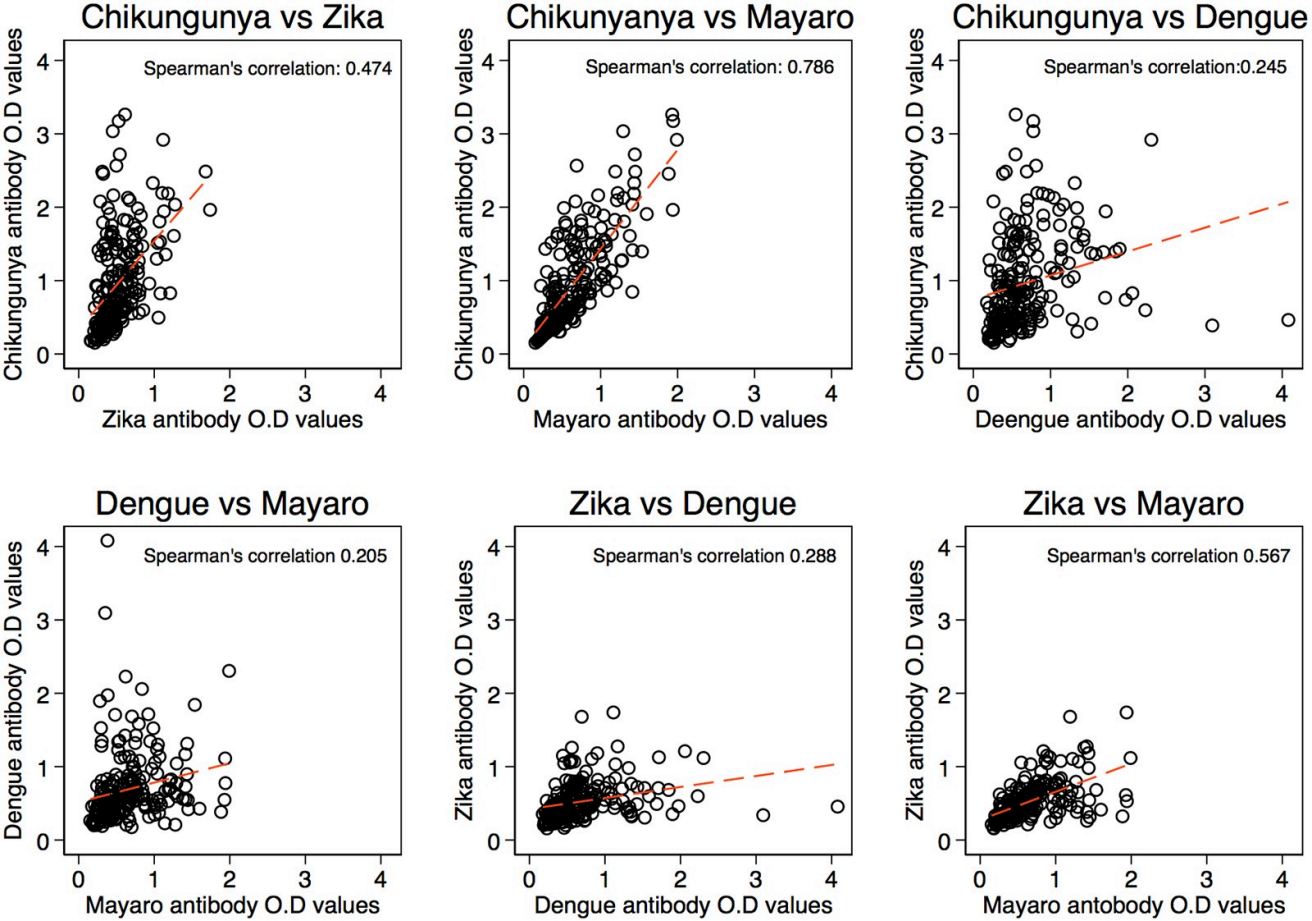
Correlations between anti-arbovirus antibodies in serum.

We also checked for multiple transmission or exposure to arboviruses among the tested sera and found that in the presence of anti-Chikungunya antibodies, the probability of antibodies to Zika (corr coeff. = 0.5) and or Mayaro (corr coeff. = 0.8) is high (Fig 4), and the least being Dengue (corr coeff. = 0.3). Similarly, if Zika was isolated, the likelihood of finding Mayaro antibodies is high (corr coeff. = 0.6) while Dengue antibodies (corr coeff. = 0.3) was low (Fig 4). If Dengue was isolated, finding Mayaro is low (corr coeff. = 0.2) (Fig 4). About 90% of the MAYV positives were found to be CHIKV positive (S1 Table).

## Discussion

We sreened individuals of high risk (mobile, cross boarder trade) and reported for the first time results on past exposure to four arboviruses in our setting-Zambia. Although no epidemics have been reported in the country, our study shows an ongoing arboviral activity in the studied population. The prevalence data on CHIKV was in line with seroprevalence studies conducted in other African countries such as Congo Brazzaville and Democratic Republic of Congo in which an estimate of 34.4% and 24.6% respectively had been reported [37,38]. However, it was far higher than that found in Tanzania [39]. The discrepency observed can be attributed to the diffecrences in study settings and or Tanzania gaining more control of the mosquito to human transmission compared to Zambia. Also, differences could be attributable to the use of different laboratory techniques and thresholds to define seropositivity.

A study by Liwewe *et al*., reported Dengue IgG seroprevalence as 4.1% from the Western and North-western regions of Zambia [40]. This data is important and is indicative of environmental suitability and continued transmission of arboviruses, given that the first case of CHIKV was reported among people involved with fishing 58 years ago on the Copperbelt Province [41]. Pierro and colleagues have also shown that antibodies against CHIKV especially the IgG last longer and in certain cases over a year post exposure [42], while a study by Nitatpattana *et al*., demonstrated the presence of CHIKV antibodies 19 years post-exposure [43]. Other researchers equally reported similar findings regarding the persistence of the antibodies in exposed individuals [44–47]. However, in our population, since no clinical data has been collected, duration of these antibodies cannot be estimated and require further investigation. Furthermore, it is unclear whether these antibodies would sufficiently protect individuals to future infections as no functional assays have equally been done.

A commentary recommending the need for more work to be done that can lead to improved arbovirus surveillance in Zambia have been published [48]. In addition, a review article by Ndashe *et al*. reported some isolated cases of congenital microcephaly in Zambia and the possibility that they could be linked to ZIKV [49]. We therefore postulate that an increase in human interaction through globalization, trade and travel coupled with the presence of *Aedes* spp in Northern and North-western province of Zambia possess greater risk of an Asian ZIKV outbreak in Zambia. Also despite the high cross border trade with back and forth movement of people to Tanzania and the Democratic Republic of Congo where major outbreaks of CHIKV have been previously reported as high [50,51], no specific efforts to track these diseases is in place.

Underpinning the lack of data is the virtually absent index of suspicion among practicing clinician for these infections. Moreover, even if suspicion was made, there remains no means of confirming diagnosis by the routine clinical laboratories in-country.

Our correlation data on the likelihood of finding exposure to more than one virus is strong for CHIKV vs MAYV as well as ZIKV vs MAYV. Potentially, for CHIKV vs MAYV, we postulate that cross reactivity for both alphavirus could be the reason of the high correlation as the proteins used were similar, with 55.3% identity (Clustal Omega alignment) and over 90% of the MAYV postives were found to be CHIKV positive. For the remaining 10% of MAYV positives that were negative for CHIKV, a possibility of cross reactivity to E2 of other alphavirus could be an explanation for the positivity. Nevertheless, one of the limitations of our study is the lack of assessment of infection during the acute phase for confirmation of the virus presence. Neutralisation assays for various alphavirus could also provide further information to support the conclusions of likelihood of cross reactivity between MAYV and CHIKV. With regards to the proteins used for ZIKV (NS1) vs MAYV (E2), these are very different and therefore, the likelihood of finding responses to an alphavirus, in this case, to MAYV E2 in a person suspected to have ZIKV infection is high. Our observations indicate that further studies of seroprevalence, coupled with molecular detection and use of *in vitro* neutralisation assays are necessary to understand virus transmission and support preparedness for potential outbreaks.

We also explored the use of saliva a less invasive test and noted a rather weaker correlation statistically compared to what has been reported elsewhere [52,53]. This difference maybe associated with the differences in sample collection time points as well as saliva being a little more dilute than serum. However, we believe that saliva can still be considered for future testing during acute infection especially in younger children. After exploring virus synergism and or co-transmissions, it is apparent that co-infections are likewise common in Zambia as reported elsewhere [54] and require tests that are highly sensitive and specific to clearly detect individual viruses during infection. In addition, numerous important questions remain to be answered though. For instance, it remains unknown whether the presence of multiple infecting arboviruses within a patient impacts short-and/or long-term clinical outcomes. Cohort studies adequately powered would be urgently required in order to fully evaluate the clinical implications of co-infection and to understand the possible impact on congenital disease.

The study has some limitations worth mentioning. Because we tested for IgG antibodies, we were not able to clearly determine disease burden report on current infection. That is, we did not include kits to test for IgM. Thus, we might be missing out on serological indications from infections that might have taken place in recent past. The timing of saliva and serum collection were not matched during testing as saliva was only collected at one time point (one year) in the parent study. Since we did not collect and speciate mosquitoes, we cannot firmly conclude on the presence of arboviruses in central province. Future studies should include children in order to report on correlation of the arboviruses to the recently reported cases of microcephaly in Zambia as well participants with fever as this has been recorded among the main complaints of patients diagnosed with arboviral infection [39]. Finally, an important consideration is that we were not able to run RT-PCR or sequencing assays to report on actual virus presence or spread of MAYV to other parts of Africa, as we have considered that MAYV circulation has only been reported in the Americas [55,56].

## Conclusion

Our findings indicate that the use of simple tecnhiques for sero-prevalence studies can provide an initial view on the exposure to arboviruses by humans and circulation in endemic regions in Zambia. We consider that there is a need to strengthen surveillance activities on arboviral infections in the country applying further techniques to deepen our understanding of the epidemiology of arboviruses circulating in humans and ultimately strengthening clinical support to infected people. As *Aedes aegypti* mosquitoes have clearly spread to many parts of Africa including Zambia, our study highlights the importance to set up routine diagnostic capacities countrywide.

## Recommendation

An urgent need remains for improved surveillance in Zambia for tracking active infection and source for development of better control and prevention strategies. Consequently, this would link some cases of congenital microcephaly to specific arboviruses. Future studies should also consider including infants and children under 5 to assess for possible transmission of the viruses during pregnancy from infected mothers especially for countries with a high burden of CHIKV.

## Supporting information

S1 Table Sample positive for CHIKV and MAYV. (DOCX)

## References

[1] Esposito Danillo Lucas Alves; Da Fonseca BAL. Complete Genome Sequence of Mayaro Virus (Togaviridae,. Genome Announc 2015;3:141660 10.1128/genomeA.01372-15 Copyright. 26679574 PMC4683219

[2] Alva-Urcia C, Aguilar-Luis MA, Palomares-Reyes C, Silva-Caso W, Suarez-Ognio L, Weilg P, et al Emerging and reemerging arboviruses: A new threat in Eastern Peru. PLoS One 2017;12:1–13. 10.1371/journal.pone.0187897 29136650 PMC5685628

[3] Brasileira S, Tropical DM, Marcondes CB, De Fátima M, De Melo F. Zika virus in Brazil and the danger of infestation by Aedes (Stegomyia) mosquitoes 2016;49:4–10. 10.1590/0037-8682-0220-2015 26689277

[4] Weetman D, Kamgang B, Badolo A, Moyes CL, Shearer FM, Coulibaly M, et al Aedes Mosquitoes and Aedes-Borne Arboviruses in Africa: Current and Future Threats. Int J Environ Res Public Health 2018;15:220 10.3390/ijerph15020220 29382107 PMC5858289

[5] Noor R, Ahmed T. Zika virus: Epidemiological study and its association with public health risk. J Infect Public Health 2018;11:611–6. 10.1016/j.jiph.2018.04.007 29706319

[6] Ganesan VK, Duan B, Reid SP. Chikungunya virus: Pathophysiology, mechanism, and modeling. Viruses 2017;9:1–14. 10.3390/v9120368 29194359 PMC5744143

[7] Mota MT de O, Terzian AC, Silva MLCR, Estofolete C, Nogueira ML. Mosquito-transmitted viruses–the great Brazilian challenge. Brazilian J Microbiol 2016;47:38–50. 10.1016/j.bjm.2016.10.008 27818091 PMC5156505

[8] Brustolin M, Pujhari S, Henderson CA, Rasgon JL. Anopheles mosquitoes may drive invasion and transmission of Mayaro virus across geographically diverse regions. PLoS Negl Trop Dis 2018;12:1–11. 10.1371/journal.pntd.0006895 30403665 PMC6242690

[9] Kantor AM, Lin J, Wang A, Thompson DC, Franz AWE. Infection Pattern of Mayaro Virus in Aedes aegypti (Diptera: Culicidae) and Transmission Potential of the Virus in Mixed Infections With Chikungunya Virus. J Med Entomol 2019;56:832–43. 10.1093/jme/tjy241 30668762 PMC6467640

[10] Jamali Moghadam SR, Bayrami S, Jamali Moghadam S, Golrokhi R, Golsoorat Pahlaviani F, SeyedAlinaghi SA. Zika virus: A review of literature. Asian Pac J Trop Biomed 2016;6:989–94. 10.1016/j.apjtb.2016.09.007

[11] Paixão ES, Barreto F, Da Glória Teixeira M, Da Conceição N Costa M, Rodrigues LC. History, epidemiology, and clinical manifestations of Zika: A systematic review. Am J Public Health 2016;106:606–12. 10.2105/AJPH.2016.303112 26959260 PMC4816002

[12] World Health Organisation (WHO). WHO statement on the first meeting of the International Health Regulations (IHR 2005) Emergency Committee on Zika virus and observed increase in neurological disorders and neonatal malformations: World Health Organization (2016). https://www.who.int/news-room/detail/01-02-2016-who-statement-on-the-first-meeting-of-the-international-health-regulations-(2005)-(ihr-2005)-emergency-committee-on-zika-virus-and-observed-increase-in-neurological-disorders-and-neonatal-malformations (accessed 19 December 2019).

[13] Schuler-faccini L, Ribeiro EM, Feitosa IML, Horovitz DDG, Cavalcanti DP. Possible Association Between Zika Virus Infection and Microcephaly—2016;65:59–62. 10.15585/mmwr.mm6503e2 26820244

[14] Cauchemez S. Besnard M., Bompard P., Dub T., Guillemette-Artur P., Eyrolle-Guignot D. et al (2016) Association between Zika virus and microcephaly in French Polynesia, 2013–15: A retrospective study. Lancet 387:2125–2132. 10.1016/S0140-6736(16)00651-6 26993883 PMC4909533

[15] Kleber de Oliveira W., Cortez-Escalante J., Gonçalves Holanda De Oliveira WT, Ikeda do Carmo GM, Pessanha Henriques CM, Coelho GE, Araújo de França GV. Increase in Reported Prevalence of Microcephaly in Infants Born to Women Living in Areas With Confirmed Zika Virus Transmission During the First Trimester of Pregnancy—Brazil, 2015. MMWR Morb Mortal Wkly Rep. 2016 3 11;65(9):242–7. 10.15585/mmwr.mm6509e2 26963593

[16] Brasil P, Pereira JP, Moreira ME, Ribeiro Nogueira RM, Damasceno L, et al Zika virus infection in pregnant women in Rio de Janeiro. N Engl J Med 2016; 375:2321–2334. 10.1056/NEJMoa1602412 26943629 PMC5323261

[17] Mlakar J, Korva M, Tul N, Popović M, Poljšak-Prijatelj M, Mraz J, et al Zika Virus Associated with Microcephaly N Engl J Med 2016; 374:951–958. 10.1056/NEJMoa1600651 26862926

[18] Heukelbach J, Alencar CH, Kelvin AA, de Oliveira WK, Pamplona de Góes Cavalcanti L (2016) Zika virus outbreak in Brazil. J Infect Dev Ctries 10:116–120. 10.3855/jidc.8217 26927450

[19] Rasmussen SA, Jamieson DJ, Honein MA, Petersen LR. Zika Virus and Birth Defects—Reviewing the Evidence for Causality. N Engl J Med: 2016; 374:1981–1987 10.1056/NEJMsr1604338 27074377

[20] Krauer F, Riesen M, Reveiz L, Oladapo OT, Martínez-Vega R, Porgo TV, et al Zika Virus Infection as a Cause of Congenital Brain Abnormalities and Guillain-Barré Syndrome: Systematic Review. PLoS Med 2017;14:e1002203–e1002203. 10.1371/journal.pmed.1002203 28045901 PMC5207634

[21] Medina MT, Medina-Montoya M. New spectrum of the neurologic consequences of Zika. J Neurol Sci 2017;383:214–5. 10.1016/j.jns.2017.10.046 29108750

[22] Kahamba NF, Limwagu AJ, Mapua SA, Msugupakulya BJ, Msaky DS, Kaindoa EW, et al Habitat characteristics and insecticide susceptibility of Aedes aegypti in the Ifakara area, south-eastern Tanzania. Parasit Vectors 2020;13:53 10.1186/s13071-020-3920-y 32033619 PMC7006121

[23] Mbanzulu KM, Mboera LEG, Luzolo FK, Wumba R, Misinzo G, Kimera SI. Mosquito—borne viral diseases in the Democratic Republic of the Congo: a review. Parasit Vectors 2020:1–11 32103776 10.1186/s13071-020-3985-7 PMC7045448

[24] Masaninga F, Muleba M, Masendu H, Songolo P, Mweene-Ndumba I, Mazaba-Liwewe ML, et al Distribution of yellow fever vectors in Northwestern and Western Provinces, Zambia. Asian Pac J Trop Med 2014;7:S88–92. 10.1016/S1995-7645(14)60210-8 25312199

[25] Mazaba-Liwewe ML, Siziya S, Monze M, Mweene-Ndumba I, Masaninga F, Songolo P, et al First sero-prevalence of dengue fever specific immunoglobulin G antibodies in Western and North-Western provinces of Zambia: A population based cross sectional study. Virol J 2014;11:1–7 25078113 10.1186/1743-422X-11-135 PMC4127398

[26] Masaninga F, Chanda E, Chanda-Kapata P, Hamainza B, Masendu HT, Kamuliwo M, et al Review of the malaria epidemiology and trends in Zambia. Asian Pac J Trop Biomed 2013;3:89–94. 10.1016/S2221-1691(13)60030-1 23593585 PMC3627166

[27] Raut CG, Rao NM, Sinha DP, Hanumaiah H, Manjunatha MJ. Chikungunya, Dengue, and Malaria Co-Infection after Travel to Nigeria, India. Emerg Infect Dis 2015;21:908–9. 10.3201/eid2105.141804 25898147 PMC4412233

[28] Ayorinde AF, Oyeyiga AM, Nosegbe NO, Folarin OA. A survey of malaria and some arboviral infections among suspected febrile patients visiting a health centre in Simawa, Ogun State, Nigeria. J Infect Public Health 2016;9:52–9. 10.1016/j.jiph.2015.06.009 26256113

[29] Oliveira Melo AS, Malinger G, Ximenes R, Szejnfeld PO, Alves Sampaio S, Bispo de Filippis AM. Zika virus intrauterine infection causes fetal brain abnormality and microcephaly: tip of the iceberg? Ultrasound Obstet Gynecol 2016;47:6–7. 10.1002/uog.15831 26731034

[30] Rodriguez-Morales AJ. Zika and microcephaly in Latin America: An emerging threat for pregnant travelers? Travel Med Infect Dis 2016;14:5–6. 10.1016/j.tmaid.2016.01.011 26879565

[31] Heymann DL, Hodgson A, Sall AA, Freedman DO, Staples JE, Althabe F, et al Zika virus and microcephaly: why is this situation a PHEIC? Lancet 2016;387:719–21. 10.1016/S0140-6736(16)00320-2 26876373 PMC7134564

[32] Central statistics office. 2010 Census of population and housing. Natl Anal Rep 2012:1–117.

[33] Chabwela H, Chomba C, Thole L. The Habitat Structure of Lukanga Ramsar Site in Central Zambia: An Understanding of Wetland Ecological Condition 2017:406–32. 10.4236/oje.2017.76029

[34] Kim YC, L C, Garcia-larragoiti N, Cano-mendez A, Hernandez-flores KG, Viveros-sandoval ME, et al. Development of an E2 ELISA Methodology to Assess Chikungunya Seroprevalence in Patients from an Endemic Region of Mexico 2019. 10.3390/v11050407 PMC6563309 31052472

[35] Kim YC, Lopez-camacho C, Nettleship JE, Rahman N, Hill ML, Silva-reyes L, et al Optimization of Zika virus envelope protein production for ELISA and correlation of antibody titers with virus neutralization in Mexican patients from an arbovirus endemic region 2018:1–12. 10.1186/s12985-018-1104-6 PMC6307127 30587198

[36] Kajeguka DC, Kaaya RD, Mwakalinga S, Ndossi R, Ndaro A, Chilongola JO, et al Prevalence of dengue and chikungunya virus infections in north-eastern Tanzania: a cross sectional study among participants presenting with malaria-like symptoms. BMC Infect Dis 2016;16:183 10.1186/s12879-016-1511-5 27112553 PMC4845349

[37] Proesmans S, Katshongo F, Milambu J, Fungula B, Muhindo Mavoko H, Ahuka-Mundeke S, et al Dengue and Chikungunya among Febrile Outpatients in Kinshasa, Democratic Republic of Congo: a cross-sectional study. BioRxiv 2018:486407. 10.1101/486407 PMC6748445 31487279

[38] Moyen N, Thiberville S-D, Pastorino B, Nougairede A, Thirion L, Mombouli J-V, et al First Reported Chikungunya Fever Outbreak in the Republic of Congo, 2011. PLoS One 2014;9:e115938 10.1371/journal.pone.0115938 25541718 PMC4277398

[39] Kinimi E, Shayo MJ, Patrick BN, Angwenyi SO, Kasanga CJ, Weyer J, et al Evidence of chikungunya virus infection among febrile patients seeking healthcare in selected districts of Tanzania. Infect Ecol Epidemiol 2018;8:1553460 10.1080/20008686.2018.1553460 30834070 PMC6394322

[40] Mazaba-liwewe ML, Siziya S, Monze M, Mweene-ndumba I, Masaninga F. First sero-prevalence of dengue fever specific immunoglobulin G antibodies in Western and North-Western provinces of Zambia: a population based cross sectional study. 2014;11:1–7. 10.1186/1743-422X-11-135 25078113 PMC4127398

[41] RODGER LM. An outbreak of suspected Chikungunya fever in Northern Rhodesia. S Afr Med J 1961;35:126–8. 13742532

[42] Pierro A, Rossini G, Gaibani P, Finarelli AC, Moro ML, Landini MP, et al Persistence of anti–chikungunya virus–specific antibodies in a cohort of patients followed from the acute phase of infection after the 2007 outbreak in Italy. New Microbes New Infect 2015;7:23–5. 10.1016/j.nmni.2015.04.002 26106482 PMC4475829

[43] Nitatpattana N, Kanjanopas K, Yoksan S, Satimai W, Vongba N, Langdatsuwan S, et al Long-term persistence of Chikungunya virus neutralizing antibodies in human populations of North Eastern Thailand. Virol J 2014;11:183 10.1186/1743-422X-11-183 25330992 PMC4283153

[44] Couderc T, Khandoudi N, Grandadam M, Visse C, Gangneux N, Bagot S, et al Prophylaxis and Therapy for Chikungunya Virus Infection. J Infect Dis 2009;200:516–23. 10.1086/600381 19572805 PMC7109959

[45] Kam Y-W, Simarmata D, Chow A, Her Z, Teng T-S, Ong EKS, et al Early Appearance of Neutralizing Immunoglobulin G3 Antibodies Is Associated With Chikungunya Virus Clearance and Long-term Clinical Protection. J Infect Dis 2012;205:1147–54. 10.1093/infdis/jis033 22389226 PMC3295607

[46] Tandale BV, Sathe PS, Arankalle VA, Wadia RS, Kulkarni R, Shah SV, et al Systemic involvements and fatalities during Chikungunya epidemic in India, 2006. J Clin Virol 2018;46:145–9. 10.1016/j.jcv.2009.06.027 19640780

[47] Theamboonlers A, Rianthavorn P, Praianantathavorn K, Wuttirattanakowit N, Poovorawan Y. Clinical and molecular characterization of Chikungunya virus in south Thailand. Jpn J Infect Dis 2009;62:303–5. 19628911

[48] Munsaka S. Zika Virus: Why Should We Care? What Do We Do About It? J Prev Rehabil Med 2016;1:4–6.

[49] Ndashe K, Nalondwa N, Khondowe O, Munjita S. Journal of Preventive and Rehabilitative Medicine Review Paper Zika Virus and Congenital Microcephaly in Zambia, What are the Chances? 2016;1:35–40.

[50] Makiala-Mandanda S, Ahuka-Mundeke S, Abbate JL, Pukuta-Simbu E, Nsio-Mbeta J, Berthet N, et al Identification of Dengue and Chikungunya Cases Among Suspected Cases of Yellow Fever in the Democratic Republic of the Congo. Vector-Borne Zoonotic Dis 2018;18:364–70. 10.1089/vbz.2017.2176 29768102

[51] Zeller H, Van Bortel W, Sudre B. Chikungunya: Its History in Africa and Asia and Its Spread to New Regions in 2013–2014. J Infect Dis 2016;214:S436–40. 10.1093/infdis/jiw391 27920169

[52] Cuzzubbo AJ, Vaughn DW, Nisalak A, Suntayakorn S, Aaskov J, Devine PL. Detection of Specific Antibodies in Saliva during Dengue Infection 1998;36:3737–9. 10.1128/jcm.36.12.3737-3739.1998 PMC105280 9817913

[53] Vázquez S, Cabezas S, Pérez AB, Pupo M, Ruiz D, Calzada N, et al Kinetics of antibodies in sera, saliva, and urine samples from adult patients with primary or secondary dengue 3 virus infections. Int J Infect Dis 2007;11:256–62. 10.1016/j.ijid.2006.05.005 16914345

[54] Magalhaes T, Robison A, Young MC, B WC Iv, Foy BD, Ebel GD, et al Sequential Infection of Aedes aegypti Mosquitoes with Chikungunya Virus and Zika Virus Enhances Early Zika Virus Transmission 2018 10.3390/insects9040177 30513725 PMC6315929

[55] Pezzi L, Diallo M, Rosa-Freitas MG, Vega-Rua A, Ng LFP, Boyer S, et al GloPID-R report on chikungunya, o’nyong-nyong and Mayaro virus, part 5: Entomological aspects. Antiviral Res 2020;174:104670 10.1016/j.antiviral.2019.104670 31812638

[56] Contopoulos-Ioannidis D, Newman-Lindsay S, Chow C, LaBeaud AD. Mother-to-child transmission of Chikungunya virus: A systematic review and meta-analysis. PLOS Neglected Tropical Diseases 2018;12:6 10.1371/journal.pntd.0006510 PMC6075784 29897898

