# Supplementary material for: Sero-prevalence of arthropod-borne viral infections among Lukanga swamp residents in Zambia
Source: PLoS One. 2020 Jul 1;15(7):e0235322. doi: 10.1371/journal.pone.0235322 (PMC7329080; doi:10.1371/journal.pone.0235322)
Supplement: S1 Table — (DOCX) [file pone.0235322.s001.docx]

Supplementary 1: Sample Positive for CHIKV and MAYV

| Patient ID | CHIKV | MAYV | Double Positive |
| --- | --- | --- | --- |
| 4 | 2.9185 | 1.991 | 1 |
| 5 | 2.0355 | 1.4115 | 1 |
| 7 | 2.329 | 1.436 | 1 |
| 9 | 1.964 | 1.9385 | 1 |
| 14 | 1.238 | 1.0355 | 1 |
| 16 | 3.2625 | 1.93 | 1 |
| 17 | 1.3745 | 0.9875 | 1 |
| 25 | 1.47 | 1.0695 | 1 |
| 30 | 0.864 | 1.0885 | 0 |
| 44 | 2.1935 | 1.2225 | 1 |
| 45 | 1.1005 | 1.009 | 1 |
| 47 | 2.094 | 1.2115 | 1 |
| 50 | 3.174 | 1.9425 | 1 |
| 51 | 1.8865 | 1.0395 | 1 |
| 52 | 0.846 | 1.4125 | 0 |
| 54 | 0.974 | 1.1965 | 0 |
| 60 | 3.0355 | 1.293 | 1 |
| 67 | 2.128 | 1.297 | 1 |
| 72 | 2.484 | 1.45 | 1 |
| 73 | 1.8285 | 1.186 | 1 |
| 75 | 1.3965 | 1.537 | 1 |
| 76 | 1.805 | 1.305 | 1 |
| 77 | 1.6305 | 1.1455 | 1 |
| 90 | 1.6095 | 1.379 | 1 |
| 91 | 1.5555 | 1.0485 | 1 |
| 103 | 1.4915 | 0.977 | 1 |
| 106 | 1.3355 | 1.003 | 1 |
| 125 | 1.5305 | 1.066 | 1 |
| 127 | 2.4555 | 1.8845 | 1 |
| 132 | 1.907 | 1.5995 | 1 |
| 133 | 2.1855 | 1.4335 | 1 |
| 147 | 2.1625 | 0.968 | 1 |
| 166 | 1.7155 | 1.0055 | 1 |
| 179 | 2.4855 | 1.191 | 1 |
| 182 | 1.415 | 1.415 | 1 |
| 184 | 1.269 | 1.269 | 1 |
| 193 | 1.063 | 1.063 | 1 |
| 199 | 1.1395 | 1.1395 | 1 |
| 201 | 1.279 | 1.279 | 1 |
| 214 | 1.048 | 1.048 | 1 |
| 219 | 1.0055 | 1.0055 | 0 |
| 221 | 2.719 | 1.444 | 1 |

38/42(90.5%) are double positive and 4/42 (9.5%) are positive for MAYV only
